# Influence of the COVID-19 pandemic on chronic disease management among indigenous people in Canada

**DOI:** 10.3389/fpubh.2026.1741996

**Published:** 2026-02-18

**Authors:** Cerina Dubois, Allison Soprovich, Lisa A. Wozniak, Lynden Crowshoe, Lea Bill, Bonnie Healy, Jeanette Jackson, Salim Samanani, Dean T. Eurich

**Affiliations:** 1 School of Public Health, 2–040 Li Ka Shing Centre for Health Research Innovation, University of Alberta, Edmonton, AB, Canada; 2 Department of Mental Health, Bloomberg School of Public Health, Johns Hopkins University, Baltimore, MD, United States; 3 Alliance for Canadian Health Outcomes Research in Diabetes, 2–040 Li Ka Shing Centre for Health Research Innovation, University of Alberta, Edmonton, AB, Canada; 4 Department of Family Medicine, Cumming School of Medicine, University of Calgary, Calgary, AB, Canada; 5 The Alberta First Nations Information Governance Centre, Calgary, AB, Canada; 6 Blackfoot Confederacy Tribal Council, Calgary, AB, Canada; 7 Health Quality Alberta, Calgary, AB, Canada; 8 OKAKI Health Intelligence Inc., Calgary, AB, Canada

**Keywords:** chronic disease, COVID-19, First Nations, healthcare utilization, indigenous

## Abstract

**Introduction:**

In Canada, little is known about how the Coronavirus-19 (COVID-19) pandemic has influenced Indigenous people with chronic disease. Even without the additional burden of the pandemic, many Indigenous communities lack access to high-quality health care and preventive measures. This study examines the impact of the COVID-19 pandemic on chronic disease management (CDM) and healthcare access and utilization by describing the CDM experiences of the Indigenous people across Canada.

**Methods:**

In collaboration with Indigenous leadership, we conducted an online survey across Canada. Eligible participants included Indigenous people (on- and off-reserve), who are current members of a Dynata research panel and willing to participate in the survey. Questions on self efficacy, healthcare utilization, and COVID-19 impact on healthcare access and utilization were asked. Descriptive statistics and multivariable regression were used.

**Results:**

There were 869 respondents in which 60% were females (*N* = 520) and the majority lived off-reserve (*N* = 657, 76%). The health condition with the highest frequency in this cohort of respondents was ‘mental illness’ (*N* = 397, 46%). Overall, 79% of respondents reported delaying chronic disease care during the COVID-19 peak. When comparing peak versus post COVID-19, the category of ‘access,’ specifically ‘waiting time too long’ (63.6% (peak) and 58.4% (post)) was the most frequently reported reason for not accessing healthcare (*p* < 0.001). Trends were similar regardless of the number of health conditions and reserve status.

**Discussion:**

Our survey on chronic disease management during peak and post-COVID-19 revealed that, while some COVID-19 related barriers improved post-pandemic, persistent challenges in healthcare access remain for Indigenous peoples in Canada.

## 1 Introduction

The Coronavirus-19 (COVID-19) pandemic brought about significant changes in chronic disease management (CDM) for those seeking care for non-COVID-19 conditions, including diabetes, heart disease, and mental illness (1, 2). While healthcare delivery systems adapted in response to the pandemic, the shift of health resources to focus on COVID-19 may have potentially affected the diagnosis, treatment, and control of chronic disease (3, 4). Internationally (5), the pandemic demonstrated a harsh dichotomy between regions and peoples. Uncontrolled entry of outsiders exacerbated the crisis for groups like the Yanomami (Amazon Basin) although conversely Indigenous communities in Australia (6) and New Zealand successfully asserted self-governance by implementing local blockades and culturally-safe health responses, resulting in proportionately lower case rates than in other regions.

Indeed, Indigenous communities in Canada demonstrated exceptional resilience during the COVID-19 pandemic by rapidly adapting community supports and programs to meet the evolving needs of their members. Through proactive public health measures, culturally-relevant interventions, and strong local leadership, they were able to implement swift responses, including vaccination campaigns, food security initiatives, and mental health supports, often outpacing broader provincial and national efforts. Despite these supportive measures, studies have shown that this shift may have disproportionately impacted Indigenous peoples in Canada, as a substantial number of First Nations, *Métis, and Inuit* communities continue to experience significant ongoing pressures due to a lack of access to high-quality healthcare, essential services, and other preventive measures (7). Collectively, health, social, and systemic inequities; discrimination and racism; isolation and inability to travel due to the lack of resources or travel restrictions (8); and overall shortages in the basic social determinants of health created a ‘perfect storm’ within Indigenous communities in Canada to experience detrimental indirect consequences of COVID-19 (9, 10).

The COVID-19 pandemic underscored substantial deficiencies in Canada’s public health surveillance and response infrastructure, particularly in relation to Indigenous communities (11, 12). For example, First Nations individuals were more likely to report unmet health care needs, disruptions in appointments, and delays in receiving essential services such as chronic disease management, screening, and mental health support (13). Moreover, heightened symptoms of anxiety, depression, and overall declines in mental well-being among Indigenous peoples has also been reported (10). Canadian Government reports have noted that First Nations people reported more unmet health needs, service delays, and disruptions in chronic disease management, with 21% of those living off-reserve experiencing unmet health care needs compared to 15% of non-Indigenous people. Despite remarkable resilience throughout the pandemic by Indigenous communities, comprehensive data on how these communities have navigated healthcare services post-pandemic (since the peak of the pandemic) is extremely limited (13, 14).

There is an urgent need to understand healthcare patterns, including adoption of virtual health, access behaviours, and how Indigenous people adapted in their CDM post COVID-19 compared to during peak COVID-19 (15). Characterization of during peak and post-pandemic health services utilization behaviours for CDM will assist in developing future strategies and policies that can improve healthcare delivery and equity for Indigenous populations (16). To fill this evidence gap, we conducted a cross-sectional study using an online survey to describe Indigenous peoples’ experiences across Canada of the impact of the COVID-19 pandemic on chronic disease management during its peak (March 2020–2022) and the period of emergence (2023–2024).

## 2 Materials and methods

### 2.1 Participants and study design

A cross-sectional online survey design was employed. While all approaches have limitations, online surveys were chosen as they reduce participant burden, are cost-effective, and allowed for a broad Canada-wide sample. The survey measures were based on a previously developed and administered survey that was co-developed in collaboration with First Nations leadership in Alberta and Health Quality Alberta. The research team, including local Indigenous representatives, finalized the survey collaboratively, ensuring the questions were relevant to the research question and reflected culturally appropriate practices and language.

Recruiting Indigenous peoples in Canada presents substantial logistical challenges. To support adequate sampling, participants were therefore recruited through a research panel maintained by Dynata.^1^ Dynata’s global network includes Canadian panelists enrolled through multiple recruitment strategies, such as organizational websites, social media, and direct email within consumer brand loyalty programs. This approach facilitated access to a specialized and geographically dispersed population and provided efficiencies in both time and cost when recruiting a large, diverse, and traditionally hard-to-reach sample (17). Importantly, feasibility considerations were balanced with methodological rigor. Dynata employs advanced fraud-detection and verification systems to ensure authentic participation and prevent duplicate entries. Eligible participants were existing members of the Dynata panel who consented to take part in the survey. In addition, participants self-identified as Indigenous Canadians (on- and off-reserve), with one or more chronic conditions, and over 18 years old. The researchers did not have access to the panels of eligible participants or distribution lists. Prior to launching the survey for planning purposes, Dynata provided an estimate of over 500 panelists representing Indigenous Canadian adults with one or more chronic conditions, with an approximate field implementation time of 21 days to gather responses.

### 2.2 Data collection

The survey was programmed into Qualtrics (18) by the research team and shared with Dynata for quality assurance and pilot testing. An invitation to the study was emailed to eligible panelists, with the Qualtrics survey link. Participants had the opportunity to review the information letter and provided written consent to participate. No paper versions were available. Participants were allowed to save and return to the survey later. Participants were awarded on a point-based system by Dynata, which may be redeemed for gift cards, airline miles or other prizes. Dynata’s comprehensive panels and points-based incentive system enhances participation rates and ensures a diverse pool of respondents (19). Survey data was collected between April 10 and May 13, 2024. In accordance with the Declaration of Helsinki, the study was approved by the University of Alberta’s Health Research Ethics Board (Study ID Pro00048714).

### 2.3 Survey design and measures

The survey was categorized into 4 general areas:

1) Basic Demographics: descriptive measures of the respondents’ Indigenous status (e.g., First Nation, Metis) age, sex/gender, location of on- or off-reserve, and types of health services available in their community (e.g., physician clinics, pharmacies, health centers).
2) General Health Questions: to capture the overall general health of the participants, the EQ-5D-5L was used. The EQ-5D-5L (20) is a standardized measure of health-related quality of life that assesses five domains: mobility, self-care, usual activities, pain/discomfort, and anxiety/depression. Each domain is rated on a 5-level scale (no problems, slight problems, moderate problems, severe problems, and extreme problems). The responses generate a health state profile, which can be converted into a single index score using Canadian-specific value sets, typically ranging from 0 (death) to 1 (full health), with some states considered worse than death (<0). Additionally, the EQ-5D-5L includes a visual analogue scale (VAS), which was also used where individuals rate their overall health from 0 (worst imaginable health) to 100 (best imaginable health).

Four questions comparing physical, mental, emotional and spiritual health between the peak of COVID-19 (2020–2022) and the present were asked with five response options ranging from much better to much worse. These questions were previously used in the Health Quality Alberta’s survey.

3) Chronic Disease Care and Management: a number of questions were posed to participants regarding their CDM and care. Included were questions related to the type of health conditions that individual is diagnosed with, years living with this chronic disease and how *confident the participants were in managing chronic disease(s)* on a scale of 1 (not at all confident) - 10 (totally confident), representing self-efficacy with respect to managing their conditions using the Self-Efficacy for Managing Chronic Disease (SEMCD) 6-item scale (21). The SEMCD covers the domains of self-management, including managing fatigue, physical discomfort/pain, emotional distress, other symptoms and tasks/activities needed to manage the health condition. This scale is not limited to a particular condition, rather it’s intended use is for various long-term illnesses, with many validation studies completed (22).
4) The Effects of COVID-19 on Chronic Disease Management: these questions were aimed at describing how and why participants accessed healthcare for their chronic disease(s), including behaviours such as delaying and/or avoiding care. Respondents could select multiple reasons within each category. Participants were directed to reflect on experiences and perceptions during the peak of the COVID-19 pandemic (March 2020 to 2022) and as we emerge from the COVID-19 pandemic, in the last year (2023–2024). We gathered responses about whether individuals needed healthcare for their chronic disease(s) during the peak of the COVID-19 pandemic AND post COVID-19 (as defined above), but did not receive the care they needed, representing a gap in usual CDM. Further, for those who did not receive the care they needed, reasons why the individual did not get care were explored (e.g., no health services within the vicinity, restrictions due to COVID-19, fear of public interactions).

### 2.4 Statistical analysis

Descriptive statistics using counts, frequencies, median, mean, and standard deviation were used to describe the respondents’ demographics. Missing data were acknowledged and reported using a complete case approach by question. Scales that were inherently Likert (i.e., ‘1’ to ‘5’) were treated as numeric; and we used mean or median categories to present the data. Moreover, analyses comparing those with and without specific sociodemographic characteristics were undertaken to fully characterize potential differences during the COVID-19 peak and post-COVID-19 (as defined above).

A multivariable linear regression was further conducted to examine the association between demographic and clinical covariates, including age group, sex, reserve status, number of chronic health conditions, and health conditions and their association with self-efficacy. In addition, to examine changes in access to healthcare use during versus after the COVID-19 peak period, a mixed-effects logistic regression analysis was conducted. The model included a fixed effect for time (post-COVID-19 vs. during COVID-19), random intercept for participants, and adjusted for demographic and clinical covariates, including age group, sex, reserve status, number of chronic health conditions, and type of health conditions. Coefficients, 95% confidence intervals, and *p*-values were estimated for each predictor. All analyses were conducted in Stata SE v.19.

### 2.5 Sensitivity analysis

In addition to the above analysis, two sensitivity analyses were conducted. First, we compared 137 (15.8%) who reported unspecified (type or number of conditions) health conditions versus those with specified chronic health conditions (*N* = 313, 36% with one health condition; *N* = 419, 48.2% with two or more health conditions) in their ability to acquire healthcare during the COVID-19 peak and post-pandemic. Likewise, we compared respondents living on (*N* = 181, 20.8%) and off-reserve (*N* = 657, 75.6%) in their care management during the COVID-19 peak and post-pandemic. In both of these analyses, an interaction term between time period (during COVID-19 versus post-COVID-19) and number of health conditions or reserve status was included in the mixed models.

## 3 Results

### 3.1 Baseline characteristics

Overall, there were a total of 869 respondents, of whom a slightly larger proportion were female (*N* = 520, 60%), and the majority lived off-reserve (*N* = 657, 76%) (Table 1). Participants reported a wide range of access to health services in their local community, including doctors’ offices (*N* = 662, 76%) and pharmacies (*N* = 698, 80%), with few reporting no access (*N* = 22, 3%). During the COVID-19 peak, 62% (*N* = 540) reported having access to virtual care or telehealth services.

**Table 1:** Baseline characteristics of the respondents from the impact of COVID-19 on chronic disease care survey (*n* = 869).

Characteristic	*N* (%)
	Total sample (*n* = 869, 100%)
Age groups	
18–29	169 (19.5)
30–39	158 (18.2)
40–49	184 (21.3)
50–59	162 (18.7)
60+	193 (22.2)
No response	3 (0.4)
Sex	
Male	303 (34.9)
Female	520 (59.8)
Other^a^, no response or prefer not to say	46 (5.3)
Province	
Atlantic Provinces^b^	75 (8.6)
British Columbia	125 (14.4)
Northern Territories^c^ and Prairie Provinces^d^	287 (33.1)
Ontario and Quebec	336 (38.7)
No response	46 (5.3)
Reserve status	
On-reserve	181 (21.0)
Off-reserve, prefer not to answer/no response	688 (79.6)
Health services available in local community (check all that apply)	
Doctor’s office	662 (76.2)
Pharmacy	698 (80.3)
Health Centre	556 (64.0)
Hospital	654 (75.3)
None or I do not know	22 (2.5)
Virtual care or telehealth was offered during COVID-19 peak	
Yes	540 (62.1)
No	194 (22.3)
No response	135 (15.5)
EQ5D	
EQ5D Single Value Index Score (Mean, SD) (missing *n* = 21)	0.74 (0.2)
EQ5D VAS Score (Mean, SD) (missing *n* = 16)	67.5 (18.5)
Type of health condition	
Mental illness	397 (45.7)
Hypertension/high blood pressure	235 (27.0)
Addiction or substance use disorder	158 (18.2)
Asthma	172 (19.8)
Diabetes	149 (17.2)
Other	355 (12.3)
Autoimmune conditions	99 (11.4)
Additional conditions^e^	122 (14.0)

a Other: Transgender, Non-binary (two spirited, 2QLGBT+), other.

b Atlantic Provinces: Newfoundland and Labrador, New Brunswick, Nova Scotia, Prince Edward Island.

c Northern Territories: Northwest Territories, Nunavut, Yukon.

d Prairie Provinces: Alberta, Saskatchewan, Manitoba.

e Additional conditions: COPD, cancer, heart attack, liver disease, stroke, kidney disease, dementia.

The EQ-5D-5L index mean score was 0.74 (SD 0.2) which suggests a moderate level of overall health status, indicating some health problems but generally a good quality of life. This score is lower than the average for the general Canadian population (which typically ranges around 0.83–0.90), suggesting a higher burden of health issues among the Indigenous participants (23). Likewise, an EQ-5D VAS mean score of 67.5 (on a scale of 0 to 100) (SD 18.5) reflects self-perceived health status and suggests that participants viewed their health as somewhat below optimal but not severely impaired. This score is also lower than the Canadian population average (usually around 80), further indicating poorer self-rated health (23–25).

**Figure 1:**
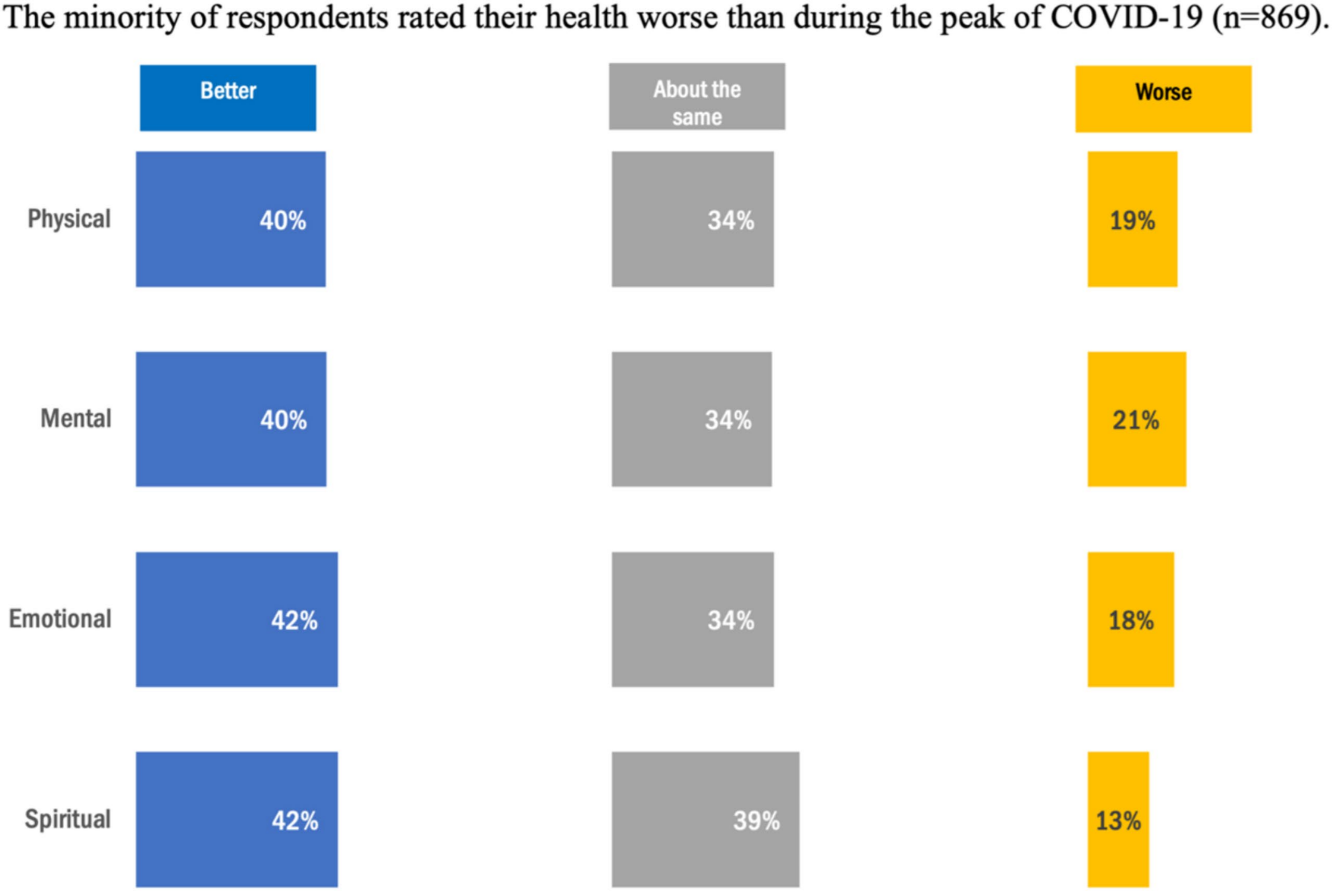
Ratings of health status comparing peak COVID-19 to post COVID-19.

**Figure 2:**
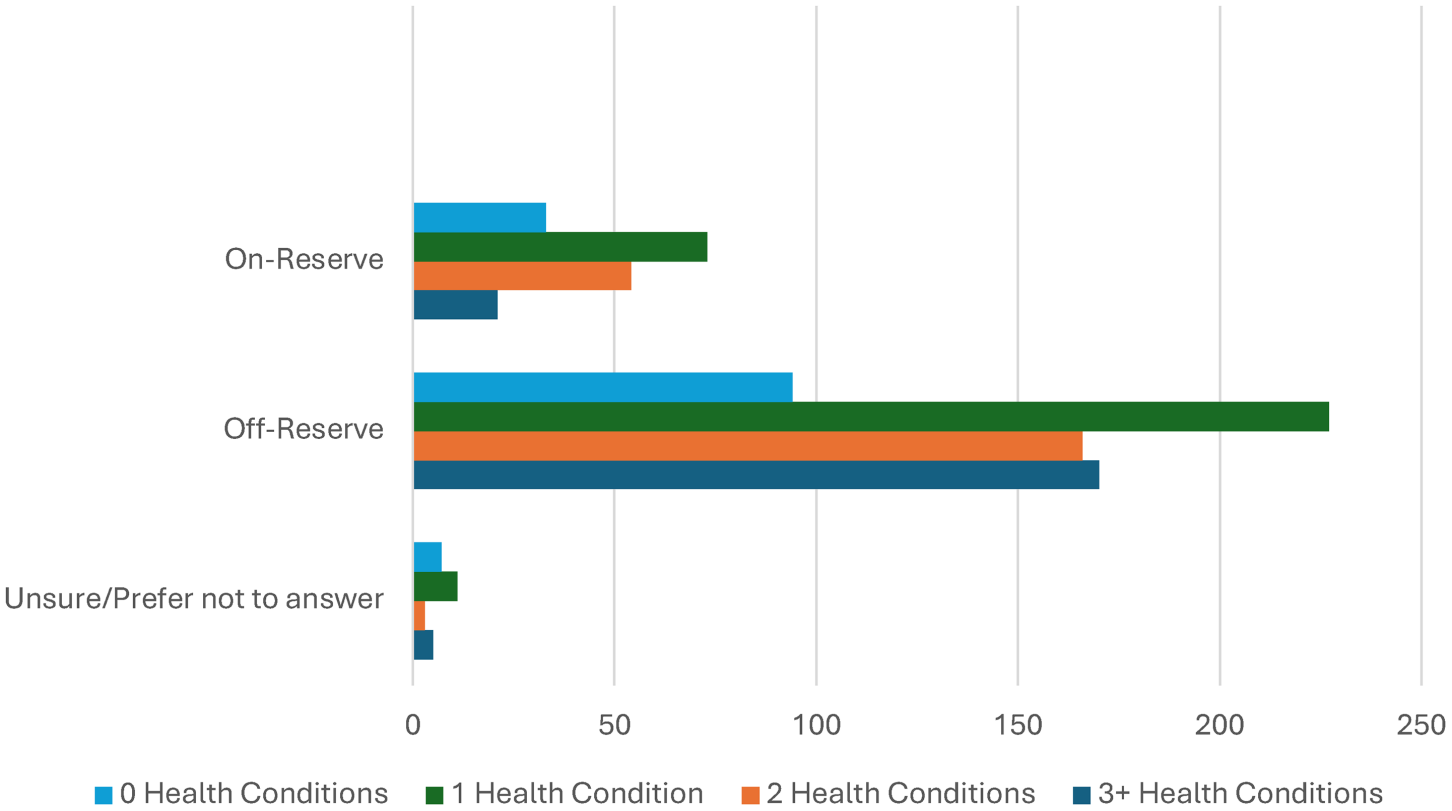
Number of health conditions by reserve status.

Overall, the health condition with the highest frequency in this cohort of respondents was ‘mental illness’ (*N* = 397, 46%). From this, 248 (63%) of respondents with ‘mental illness’ reported living with this condition for over 10 years. The second highest frequency in this cohort was ‘hypertension/high blood pressure’ (*N* = 235, 27%) with the proportion of those selecting living with this condition less than 5 years (*N* = 81, 34%), 5–9 years, (*N* = 74, 32%) and greater than 10 years (*N* = 70, 30%) (Table 1). Compared to during the peak of COVID-19, approximately 40% of respondents reported higher health ratings for physical, mental, emotional, and spiritual health (Figure 1); whereas up to 21% of respondents reported lower health ratings post-COVID. Notably, those living on-reserve (*N* = 73, 40%) were slightly more likely to report having a health condition than those living off-reserve (*N* = 227, 35%). Conversely, those living off-reserve (*N* = 170, 26%). were more likely to report having 3 health conditions than those living on-reserve (N = 21, 12%) (Figure 2).

### 3.2 Self-efficacy

Overall, the median self-efficacy scores were 6.5 (SD 2.1), suggesting that patients had medium levels of self-efficacy across their chronic diseases. There were no statistically significant differences in self-efficacy regardless of age group, sex, reserve status or years living with a health condition and were similar to the overall self-efficacy (Table 2). However, when comparing the number of health conditions, individuals who had unspecified health conditions had significantly higher median self-efficacy scores of 8.0 (SD 2.1) compared to the others (*p* < 0.0001).

**Table 2:** Median self efficacy score in managing chronic diseases by characteristic.

Characteristic	Self efficacy** Median (SD) (*N* = 809)	*p*-value
Total Cohort	6.5 (2.1)	**–**
*Age group*		0.6
18–29	6.3 (2.0)	
30–39	6.1 (2.1)	
40–49	6.2 (2.6)	
50–59	6.4 (2.0)	
60–69	6.7 (2.1)	
70+	7.5 (2.0)	
*Sex*		0.1
Male	6.9 (2.0)	
Female	6.3 (2.2)	
Other*	5.3 (2.0)	
No response or Prefer not to say	6.5 (2.6)	
*Reserve status*		0.8
On-reserve	6.5 (2.0)	
Off-reserve	6.5 (2.2)	
Unsure/prefer not to answer	6.5 (2.0)	
*Number of health conditions*		**<0.0001**
Unspecified	8.0 (2.1)	
1	6.8 (2.1)	
2	6.3 (1.8)	
3+	5.3 (2.1)	
*Years living with health condition*		0.1
<5	6.7 (2.0)	
5–9	6.7 (1.9)	
>10	6 (2.2)	

*Other: non-binary, two spirited, 2QLGPT+, or transgender.

**Self efficacy is calculated as a mean of 6 questions on the self-efficacy scale on a scale of 1 (not at all confident) to 10 (totally confident). Bolded values equate to statistically significant value *p* < 0.05.

When comparing years living with the health condition, high (8-19) self-efficacy scores were reported in individuals with <5 years of liver disease (median self-efficacy 8.2; IQR 4.7–8.3); and individuals with <5 years of asthma (median self-efficacy 8.0; IQR 6.2–8.5). The lowest self-efficacy scores (1-4) were reported in individuals with 5–9 years living with dementia (median self-efficacy 2.7). All other health conditions reported a middle level of self-efficacy (median scores between 5 and 7) (Supplementary Table 1). In multivariable models, the total number of health conditions, and a history of mental health were associated with lower self-efficacy while those with a history of asthma and hypertension reported higher self-efficacy scores. Living on or off reserve was not associated with self-efficacy, and no age or sex differences were noted in multivariable analysis (Supplementary Tables 2, 3).

### 3.3 Chronic disease care and COVID-19

**Figure 3:**
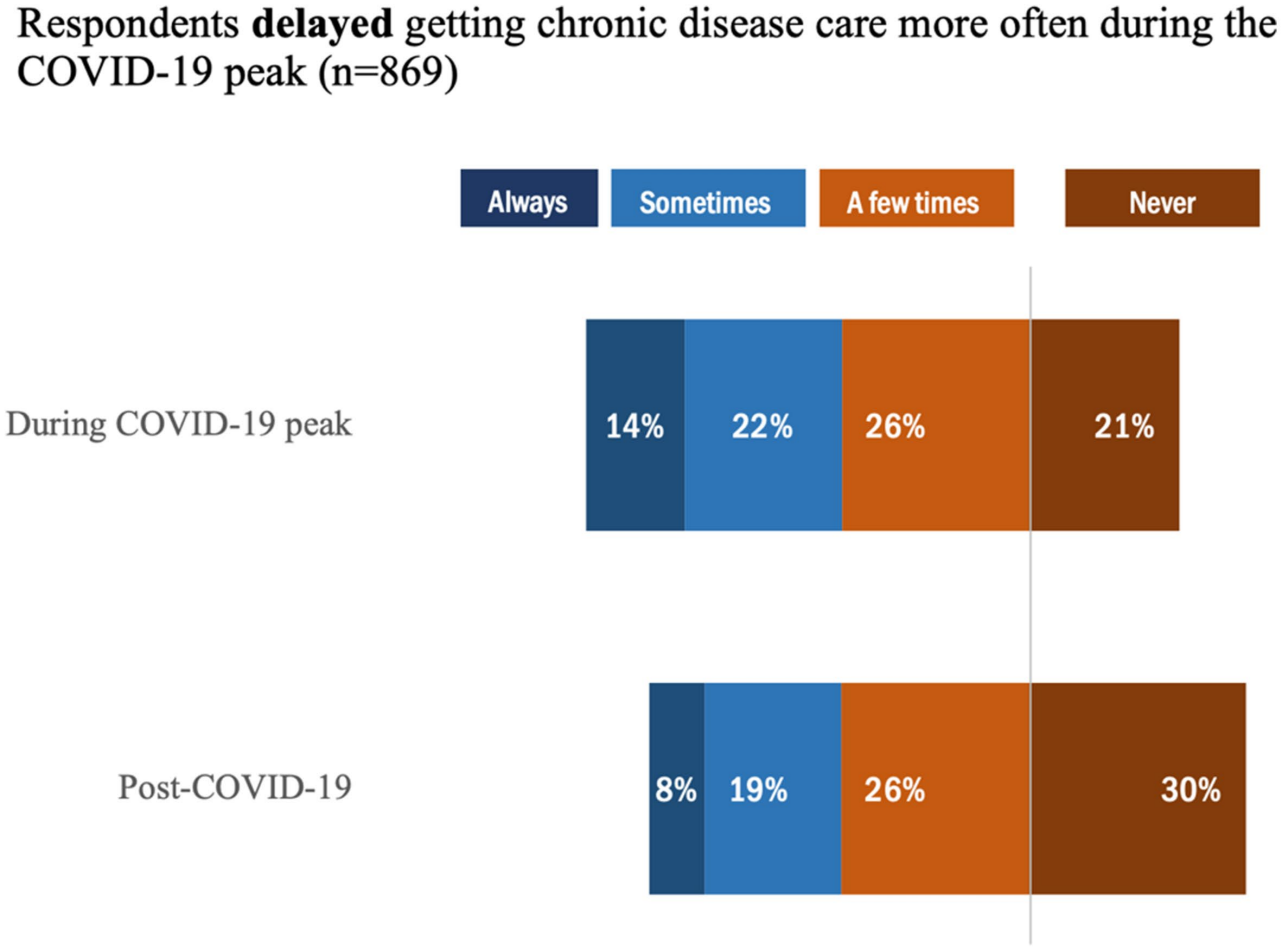
Proportion of individuals who delayed getting care during the COVID-19 peak.

**Figure 4:**
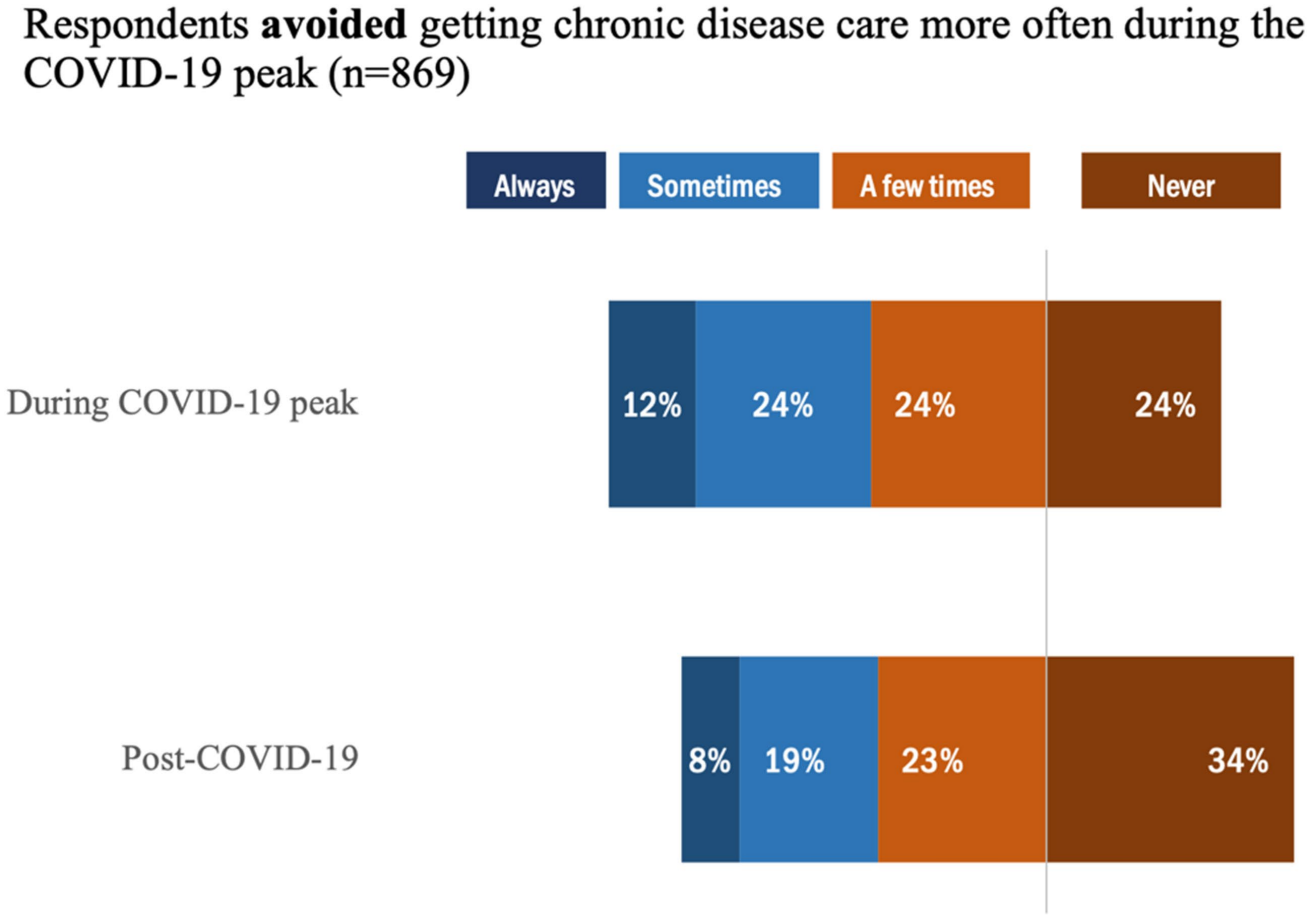
Proportion of individuals who avoided getting care during the COVID-19 peak.

Overall, 79% of respondents reported delaying chronic disease care during the COVID-19 peak, whereas this proportion dropped only slightly to 70% post-COVID-19 (Figure 3). Notably, the proportion of individuals who ‘Always’ *delayed* getting chronic disease care was higher (14%) during COVID-19 peak versus post COVID-19 (8%). Likewise, 76% of respondents reported they avoided getting chronic disease care more often during the COVID-19 peak (Figure 4) compared to 66% in the post-COVID-19 period. Similarly, for individuals who ‘Always’ *avoided* getting chronic disease care during peak COVID-19, the rate was higher (12%) versus post COVID-19 (8%); however, there was a proportional difference between those who selected ‘Never’, in which 24% indicated this during peak COVID-19 versus 34% post COVID-19.

Out of 869 respondents, 186 (21.4%) reported that they did not receive needed health care for chronic conditions during the peak of COVID-19 compared to 147 (16.9%) post COVID-19 (Table 3). The key reasons for not receiving healthcare when individuals felt they needed it (when comparing peak COVID-19 versus post COVID-19 included): concerns related to COVID-19 (52% peak; 31% post), access (69% peak; 69% post); personal reasons (41% peak; 44% post), or other (36% peak; 27% post). Although response rates were low for all categories, proportions of individuals who selected the following reasons were similar between peak COVID-19 versus post COVID-19. Notably, the highest proportion of individuals selected the category of ‘access,’ specifically ‘waiting time too long’ as a reason for not receiving healthcare [63.6% (during COVID-19) and 58.4% (post)] (Table 3).

**Table 3:** Reasons for not receiving healthcare when individuals needed it—comparing during COVID-19 peak versus post COVID-19.

Survey question*	Time period		*p*-value**
Needed health care for chronic disease(s) but did not receive it	During COVID-19 Peak (*N* = 186)	Post-COVID-19 (*N* = 147)	**<0.001**
COVID-19	97/186 (52.2)	46/147 (31.3)	**<0.001**
Fear of contracting COVID from a health practitioner/facility	55 (56.7)	31 (67.4)	
Concern of increasing burden to an already stressed health care system	35 (36.1)	19 (41.3)	
Access/ public health restrictions	55 (56.7)	17 (37.0)	
Other	6 (6.2)	3 (6.5)	
Unspecified	0	0	
Access	129/186 (69.4)	101/147 (68.7)	**<0.001**
Not available in the area	22 (17.1)	16 (15.8)	
Not available at time required	31 (24.0)	29 (28.7)	
Waiting time too long	82 (63.6)	59 (58.4)	
Felt would be inadequate	28 (21.7)	17 (16.8)	
Cost	17 (13.2)	22 (21.8)	
Personal	76/186 (40.9)	64/147 (43.5)	0.14
Didn’t get around to it/did not bother	18 (23.7)	22 (34.4)	
Decided not to seek care	21 (27.6)	17 (26.6)	
Doctor did not think it was necessary	21 (27.6)	20 (31.3)	
Personal or family responsibilities	26 (34.2)	16 (25.0)	
Dislike doctors/afraid	24 (31.6)	19 (30.0)	
Other, prefer not to answer, do not know	27 (35.5)	17 (26.6)	

*Respondents could select multiple reasons within each category.

**p-value based on mixed effects multinomial logistic regression including age group, sex, reserve status, number of chronic health conditions, and number of health conditions. Bolded values equate to statistically significant value *p* < 0.05.

Mixed effects adjusted logistic regression showed that the post-covid-19 time period showed significantly lower odds of reporting healthcare access issues due to COVID-19-related reasons (adjusted Odds ratio (aOR) = 0.22, 95% CI: 0.11–0.44, *p* < 0.001) and significantly lower odds of access-related reasons (aOR = 0.17, 95% CI: 0.08–0.39, *p* < 0.0001). However, there was no significant difference between during COVID-19 and post-COVID-19 on personal-related reasons (aOR = 0.66, 95% CI: 0.38–1.14, *p* = 0.14) (Table 4).

**Table 4:** Mixed models regression results of the association of self-efficacy on during COVID-19 versus post-COVID-19 healthcare access.

Variable	Coefficient	Lower 95% confidence interval	Upper 95% confidence interval	*p*-value
Age group	0.01	−0.05	0.07	0.82
Sex	−0.24	−0.40	−0.09	**0.003**
Reserve status	0.17	−0.06	0.41	0.14
Number of health condition group	−0.38	−0.68	−0.08	**0.01**
Mental illness	−1.0	−1.31	−0.68	**<0.0001**
Hypertension	0.25	−0.05	0.56	0.11
Addiction	−0.61	−0.96	−0.27	**<0.0001**
Asthma	023	−0.10	0.56	0.17
Diabetes	0.09	−0.27	0.44	0.62
Autoimmune conditions	−0.20	−0.58	0.18	0.29
Additional conditions*	−0.40	−0.77	−0.03	**0.03**

*Additional conditions: COPD, cancer, heart attack, liver disease, stroke, kidney disease, dementia. Bolded values equate to statistically significant value *p* < 0.05.

### 3.4 Sensitivity analyses

When stratified by the number of health conditions, the trends remained similar during peak and post-COVID-19 periods, with no statistically significant associations between health conditions and time period (*p* = 0.99) (Supplementary Table 2). Similarly, response rates were low, and the ‘access’ category was the most commonly reported reason for not receiving healthcare across all groups, regardless of the number of health conditions.

When stratified by reserve status, “waiting time” remained the most frequently reported reason for not receiving healthcare during both peak COVID-19 and the post-COVID-19 period across all groups. COVID-19–related reasons were more common during peak COVID-19 than post-COVID-19 for participants living on reserve (54% vs. 48%) and off reserve (51% vs. 27%). Patterns in “personal reasons” were also similar across groups regardless of reserve status (Supplementary Table 2). Overall, no statistically significant differences were noted between reserve status and time period (during versus post-COVID-19) (*p* = 0.99) (Supplementary Table 3).

## 4 Discussion

Overall, an online survey about CDM for Indigenous people in Canada, showed that this cohort experiences notable health challenges, which could be related to chronic conditions, social determinants of health, or access to healthcare services. Our study found that a statistically significant proportion of respondents reported avoiding or delaying healthcare during peak COVID-19 and post COVID-19. Particularly, respondents reported ‘access’ as the highest reported reason for not receiving healthcare, specifically the subcategory reason of ‘waiting times being too long’. Frequencies for all other reasons remained constant during peak versus post COVID-19 regardless of the number of health conditions reported or reserve status.

This is aligned with the literature stating access to healthcare for Indigenous people has been and continues to be a challenge due to various historical and systemic barriers (26). Globally, this stems from multifaceted barriers including geographical isolation, systemic racism and a lack of culturally safe services (27). However, 62% of respondents mentioned they had access to virtual care in their community, which may have been a safe avenue for patients to receive care for CDM (28). Knowing this, continued access and availability to virtual care may be highly beneficial for Indigenous communities going forward. Notably, Health Canada’s Equity Task Team highlighted the need for equitable access to virtual care, noting that services modeled on existing approaches can exacerbate inequities (29). Virtual healthcare must be designed and delivered in way that are culturally safe, respectful, and responsive to the diverse needs of Indigenous peoples in Canada. Some influencing factors are ensuring access to reliable telephone services and/or reliable internet for virtual visits, incorporating Indigenous languages and knowledge systems, and involving Indigenous communities in the development and implementation of virtual care services (30). Indeed, employing virtual care services without an equity-oriented framework risks widening the divide due to barriers like poor broadband access, low digital literary and concerns over data privacy (31, 32). Emphasizing access, relationships, quality and safety are essential for virtual health care to respect Indigenous self-determination and actively reduce historical health disparities.

Across both the peak COVID-19 and post-COVID-19 periods, access-related barriers, particularly waiting time, were consistently the most frequently reported reasons for unmet healthcare needs. This pattern held irrespective of reserve status, indicating that the dominant impediments to care were structural rather than geographically driven. Although COVID-19–specific disruptions declined over time, these changes did not differ meaningfully between those living on or off reserve, nor were they influenced by the number of chronic conditions reported. The absence of variation across these groups suggests that the challenges experienced were systemic in nature and not confined to particular segments of the Indigenous population. Overall, the findings highlight persistent access limitations that shaped healthcare experiences throughout the pandemic and beyond, underscoring the need for broader, system-level improvements to reduce delays and ensure more equitable access to care.

In addition, our findings that lower self-efficacy scores were correlated with higher number of health conditions is not surprising, as those with health conditions reported lower ratings in all categories of physical, emotional, spiritual, and financial categories. This is supported by literature that quality of life is highly associated with self-efficacy (33). Compounded with systemic, cultural and social factors, overall self-efficacy tends to be lower among Indigenous people, making it more challenging for individuals to engage in effective chronic disease management (34). Addressing self-efficacy here involves providing programs that build knowledge and skills to navigate the complex social and structural challenges arising from inequity, structural violence, and racism, thereby fostering critical awareness (35).

Findings from this study may help initiate a broader dialogue on how public health emergencies and ongoing access barriers shape healthcare experiences for Indigenous communities. National surveys of this kind can contribute to a broader understanding of chronic disease management and the structural challenges that influence care, particularly during periods of heightened system strain such as the COVID-19 pandemic. By demonstrating the persistence of access-related issues across time, health status, and geographic location, the results highlight the central role of system-level factors in managing chronic diseases among Indigenous people. They also help point to priority areas for future efforts, including improving timely access, reducing wait times, and strengthening continuity of services during emergencies. Collectively, this evidence can support the initial dialogue between policymakers, healthcare organizations, and Indigenous communities in developing more equitable and resilient strategies for chronic disease care moving forward.

A key strength of this study is its foundation in a prior Alberta-based survey that was co-developed with First Nations leadership, ensuring cultural relevance through meaningful collaboration. The study team included Indigenous leaders—such as First Nations community leaders, traditional knowledge holders, and Indigenous health scholars—who contributed to the cultural interpretation of the survey. Additionally, with First Nations leadership, our survey was able to capture a substantial sample of perspectives from Indigenous individuals (N = 869) across Canada, including both on-reserve and off-reserve populations. Although cross-sectional studies carry inherent methodological limitations, we believe that a study of this scale provides an important and timely contribution to understanding Indigenous health and experiences.

Nonetheless, several limitations should be acknowledged. Self-reported data and voluntary participation are subject to recall and selection bias as respondents were asked to recall experiences from 2020 to 2022. Furthermore, provincial timelines and the intensity of public health restrictions varied across Provinces and territories in Canada, which may have led to differing experiences during the COVID-19 pandemic. While the use of a membership panel platform (Dynata) facilitated broad geographic reach and recruitment efficiency, it may have introduced sampling bias and limited representativeness. Moreover, as survey questions were optional, item non-response may have led to missing data that could influence the findings. Finally, the sample size of 869 participants may constrain the generalizability of the results to the broader Indigenous population in Canada. Specifically, as there were only five respondents from the Northern Territories. Future studies would benefit from broader sampling strategies that enhance representation from the Northern Territories. However, identifying and measuring outcomes among a diverse and geographically dispersed Indigenous population remains inherently challenging, and these logistical constraints should be carefully considered in the design of subsequent research.

## 5 Conclusion

Understanding the health experiences of Indigenous people in Canada is crucial for fostering equitable healthcare outcomes. Our survey about their CDM experiences during peak and after the COVID-19 pandemic showed that COVID-19-related reasons did show improvements post-pandemic; however, healthcare access reasons continue to be a challenge in these communities. By focusing on these specific access categories, Indigenous leaders can utilize this information to examine strategies to work towards a more equitable and effective healthcare system for Indigenous people in Canada.

## Data Availability

The data supporting the findings of this study may be made available by the corresponding author upon reasonable request.

## Footnotes

1
www.dynata.com
