# Correction: Influence of the COVID-19 pandemic on chronic disease management among indigenous people in Canada

**DOI:** 10.3389/fpubh.2026.1817733

**Published:** 2026-04-13

**Authors:** Cerina Dubois, Allison Soprovich, Lisa A. Wozniak, Lynden Crowshoe, Lea Bill, Bonnie Healy, Jeanette Jackson, Salim Samanani, Dean T. Eurich

**Affiliations:** 1 School of Public Health, 2–040 Li Ka Shing Centre for Health Research Innovation, University of Alberta, Edmonton, AB, Canada; 2 Department of Mental Health, Bloomberg School of Public Health, Johns Hopkins University, Baltimore, MD, United States; 3 Alliance for Canadian Health Outcomes Research in Diabetes, 2–040 Li Ka Shing Centre for Health Research Innovation, University of Alberta, Edmonton, AB, Canada; 4 Department of Family Medicine, Cumming School of Medicine, University of Calgary, Calgary, AB, Canada; 5 The Alberta First Nations Information Governance Centre, Calgary, AB, Canada; 6 Blackfoot Confederacy Tribal Council, Calgary, AB, Canada; 7 Health Quality Alberta, Calgary, AB, Canada; 8 OKAKI Health Intelligence Inc., Calgary, AB, Canada

**Keywords:** chronic disease, COVID-19, First Nations, healthcare utilization, indigenous

Affiliation “The Alberta First Nations Information Governance Centre, Calgary, AB, Canada” was omitted from the paper. This affiliation has now been added for authors: Lea Bill.

Affiliation “Blackfoot Confederacy Tribal Council, Calgary, AB, Canada” was omitted from the paper. This affiliation has now been added for author: Bonnie Healy.

Affiliation “Health Quality Alberta, Calgary, AB, Canada” was omitted from the paper. This affiliation has now been added for author: Jeanette Jackson.

Affiliation “OKAKI Health Intelligence Inc., Calgary, AB, Canada” was omitted from the paper. This affiliation has now been added for author: Salim Samanani.

Author Bonnie Healy was erroneously assigned to affiliation Health Quality Alberta, Calgary, AB, Canada. This affiliation has now been removed for author Bonnie Healy.

Author Jeannette Jackson was erroneously assigned to affiliation OKAKI Health Intelligence Inc., Calgary, AB, Canada. This affiliation has now been removed for author Jeannette Jackson.

Author Salim Samanani was erroneously assigned to affiliation OKAKI Health Intelligence Inc., Market Mall, Calgary, AB, Canada. This affiliation has now been removed for author Salim Samanani.

The original version of this article has been updated.

