# Supplementary material for: Influence of the COVID-19 pandemic on chronic disease management among indigenous people in Canada
Source: Front Public Health. 2026 Feb 18;14:1741996. doi: 10.3389/fpubh.2026.1741996 (PMC12959125; doi:10.3389/fpubh.2026.1741996)
Supplement: Supplementary file 1 [file Supplementary_file_1.docx]

**Impact of COVID-19 on Chronic Disease Care**

**Survey**

Thank you for participating in our study by answering the following survey. Below is some information to help you answer this survey.

This confidential survey is about how the COVID-19 pandemic impacted chronic disease care for Indigenous People in across Canada.

There are no right or wrong answers to any of the questions.

Please read the questions carefully and answer each one according to what is true for you. Please answer each question to the best of your ability.

**Section A: Demographics**

**A1. What is your age group?**

🞎 18-24

🞎 25-34

🞎 35-44

🞎 45-54

🞎 55-64

🞎 65-74

🞎 75 or older

**A2. Are you**:

🞎 Male

🞎 Female

🞎 Transgender

🞎 Non-binary (Two spirted, 2QLGBT+)

🞎 If these categories do not accurately reflect how you identify yourself, please use this space to write in your response: __________________________

🞎 Prefer not to answer

**A3. Do you currently live off-reserve?**

🞎 Yes

🞎 No

🞎 Unsure

🞎 Prefer not to answer

**A4. What are the first 3 letters of the postal code where you are living/staying** (for example, if your postal code was T7J 3W1, you would write T7J):

First 3 letters: _ _ _

**A5. What type of health services are available in your local community? Please select all that apply.**

🞎 Doctor’s office

🞎 Pharmacy

🞎 Health Centre

🞎 Hospital

🞎 None

🞎 Don’t know

**Section B: General Health Questions**

**B1. EQ-5D-5L**

**Under each heading please tick the ONE box that best describes your health TODAY.**

**MOBILITY**

🞎 I have no problems in walking about

🞎 I have slight problems in walking about

🞎 I have moderate problems in walking about

🞎 I have severe problems in walking about

🞎 I am unable to walk about

**SELF-CARE**

🞎 I have no problems in washing or dressing myself

🞎 I have slight problems in washing or dressing myself

🞎 I have moderate problems in washing or dressing myself

🞎 I have severe problems in washing or dressing myself

🞎 I am unable to wash or dress myself

**USUAL ACTIVITIES**

🞎 I have no problems in doing my usual activities

🞎 I have slight problems in doing my usual activities

🞎 I have moderate problems in doing my usual activities

🞎 I have severe problems in doing my usual activities

🞎 I am unable to do my usual activities

**PAIN/DISCOMFORT**

🞎 I have no pain or discomfort

🞎 I have slight pain or discomfort

🞎 I have moderate pain or discomfort

🞎 I have severe pain or discomfort

🞎 I have extreme pain or discomfort

**ANXIETY/DEPRESSION**

🞎 I am not anxious or depressed

🞎 I am slightly anxious or depressed

🞎 I am moderately anxious or depressed

🞎 I am severely anxious or depressed

🞎 I am extremely anxious or depressed

**B2. We would like to know how good or bad your health is TODAY**

- This scale is numbered from 0 to 100.
- 100 means the best health you can imagine.
- 0 means the worst health you can imagine.
- Mark an X on the scale to indicate how your health is TODAY.
- Now, please write the number you marked on the scale in the box below.

**Section C: Your chronic disease care and management**

*We would like to know about your experiences on how the COVID-19 pandemic affected your chronic disease care/management.*

**C1. Has a doctor ever told you that you have (had) any of the following conditions?**

*(Check all that apply)*

|  |  |
| --- | --- |
| Asthma | 🞎 |
| Cancer | 🞎 |
| COPD |  |
| Hypertension / high blood pressure | 🞎 |
| Heart attack |  |
| Stroke | 🞎 |
| Diabetes | 🞎 |
| Problems with your immune system (example, Crohn’s) | 🞎 |
| Kidney Disease | 🞎 |
| Liver Disease | 🞎 |
| Dementia |  |
| Mental illness (e.g., Anxiety/depression) | 🞎 |
| Addiction or substance abuse disorder |  |
| Other, please specify: | 🞎 |

**C2. How long have you lived with [this chronic disease(s)]?**

🞎 Less than 1 year

🞎 1-4 years

🞎 5-9 years

🞎 10 or more years

🞎 I don’t know

**Section D: Confidence managing chronic disease(s)**

*We would like to know how confident you feel managing your chronic disease(s).*

*We would like to know how confident you are in doing certain activities. For each of the following questions, please choose the number that corresponds to your confidence that you can do the tasks regularly at the present time.*

D1. How confident do you feel that you can keep the fatigue caused by your disease from interfering with the things you want to do?

- Scale: 1 (not at all confident) to 10 (totally confident)

D2. How confident do you feel that you can keep the physical discomfort or pain of your disease from interfering with the things you want to do?

- Scale: 1 (not at all confident) to 10 (totally confident)

D3. How confident do you feel that you can keep the emotional distress caused by your disease from interfering with the things you want to do?

- Scale: 1 (not at all confident) to 10 (totally confident)

D4. How confident do you feel that you can keep any other symptoms or health problems you have from interfering with the things you want to do?

- Scale: 1 (not at all confident) to 10 (totally confident)

D5. How confident do you feel that you can the different tasks and activities needed to manage your health condition so as to reduce your need to see a doctor?

- Scale: 1 (not at all confident) to 10 (totally confident)

D6. How confident do you feel that you can do things other than just taking medication to reduce how much your illness affects your everyday life?

- Scale: 1 (not at all confident) to 10 (totally confident)

**D7. We are making sure you are reading the questions. Please select three below.**

🞎 One

🞎 Two

🞎 Three

**D8. Other than yourself, how many people do you help in managing their chronic disease?**

- **No one**
- **1 person**
- **2 or more people**

**Section E: Effects of COVID-19 on Chronic Disease Care**

**E1a. During the peak of the COVID-19 pandemic (March 2020 to 2022) did you need health care for your chronic disease(s) but you didn’t receive it?**

- Yes, I needed health care, but didn’t receive it
- No, I needed health care but received it
- No, I didn’t need health care
- Prefer not to answer
- Don’t know

**E1b. As we emerge from the COVID-19 pandemic, in the last year (2023-2024) have you needed health care for your chronic diseases(s) but you didn’t receive it?**

- Yes, I needed health care, but didn’t receive it
- No, I needed health care but received it
- No, I didn’t need health care
- Prefer not to answer
- Don’t know

E1c). If YES to E1 a or 1b - Why didn’t you get care? (check all that apply)

- COVID-19 (if selected, drop down list below E1b)
- Fear of contracting COVID from a health practitioner/facility
- Concern of increasing burden to an already stressed health care system
- Access/ public health restrictions
- Other: specify
- Not available in the area
- Not available at time required (e.g., doctor on holidays, inconvenient hours)
- Waiting time too long
- Felt would be inadequate
- Cost
- Didn’t get around to it/ Didn’t bother
- Decided not to seek care
- Doctor didn’t think it was necessary
- Personal or family responsibilities
- Dislike doctors/afraid
- Other
- Prefer not to answer
- Don’t know

**E2a). Thinking back to the peak of the COVID-19 pandemic (2020-2022), how often did you delay getting care for your chronic diseases(s)**

- Always
- Sometimes
- A few times
- Never
- Not applicable
- Prefer not to answer

Don’t know

E2b) **Thinking back to the peak of the COVID-19 pandemic (2020-2022), how often did you avoid getting care for your chronic diseases(s)**

- Always
- Sometimes
- A few times
- Never
- Not applicable
- Prefer not to answer
- Don’t know

**E3a). As we emerge from the COVID-19 pandemic, in the last year (2023-2024), how often did you delay getting car for your chronic disease(s)?**

- Always
- Sometimes
- A few times
- Never
- Not applicable
- Prefer not to answer
- Don’t know

**E3b). As we emerge from the COVID-19 pandemic, in the last year (2023-2024), how often did you avoid getting car for your chronic disease(s)?**

- Always
- Sometimes
- A few times
- Never
- Not applicable
- Prefer not to answer
- Don’t know

**E4. Was virtual care (i.e. telephone call, video conferencing, text, app, etc.) an option or offered to you during the peak of the COVID-19 pandemic (2020-2022)?**

- Yes
- No
- Don’t know

**E5. Compared to during the peak of the COVID-19 pandemic (2020-2022), how would you rate your physical health in general now?**

- Much better
- Slightly better
- About the same
- Slightly worse
- Much worse

**E6. Compared to during the peak of the COVID-19 pandemic (2020-2022), how would you rate your mental health (such as feeling anxious, depressed, or irritable) now?**

- Much better
- Slightly better
- About the same
- Slightly worse
- Much worse

**E7. Compared to during the peak of the COVID-19 pandemic (2020-2022), how would you rate your emotional health in general now?**

- Much better
- Slightly better
- About the same
- Slightly worse
- Much worse

**E8. Compared to during the peak of the COVID-19 pandemic (2020-2022), how would you rate your spiritual health now?**

- Much better
- Slightly better
- About the same
- Slightly worse

**Section D: Additional comments**

**D1. Please tell us anything else you want to share about your experiences with chronic disease care/management during COVID-19.**

**_________________________**
